# Chlorogenic Acid Alleviates Experimental Asthma by Reprogramming DHA Metabolism to Inhibit Ferroptosis

**DOI:** 10.3390/ijms27114747

**Published:** 2026-05-25

**Authors:** Ping Zhou, Gulimire Abudukeremu, Zhi-Li Zhang, Jing-Yi Xu, Jian-Xuan Ji, Yun-Dan Guo, Ming-Xuan Zhang, Ling Ren, Lu-Lu Wang, Zhi-Cheng Tang, Ayidana Wotan, Xiao-Juan Rong, Cai Tie, Tian-Le Gao

**Affiliations:** 1 Institute of Materia Medica, Chinese Academy of Medical Sciences & Peking Union Medical College, Beijing 100050, China; zhouping@fuwai.com (P.Z.); gulimire@imm.ac.cn (G.A.); tianlegao@imm.ac.cn (T.-L.G.); 2 State Key Laboratory and Institute of Elemento-Organic Chemistry, College of Chemistry, Nankai University, Tianjin 300071, China; dbrh06@163.com; 3 State Key Laboratory for Fine Exploration and Intelligent Development of Coal Resources, School of Chemical and Environmental Engineering, China University of Mining and Technology-Beijing, Beijing 100083, China; 4 Institute of Medicinal Biotechnology, Chinese Academy of Medical Sciences & Peking Union Medical College, Beijing 100050, China; 5 Xinjiang Key Laboratory for Uighur Medicine, Xinjiang Institute of Material Medica, Urumqi 830004, China

**Keywords:** chlorogenic acid, asthma, ferroptosis, DHA metabolism, ALOX12, lipid peroxidation, Nrf2, GPX4

## Abstract

Asthma is a chronic inflammatory disease with limited therapeutic options, highlighting the urgent need to explore alternative mechanisms and agents. Chlorogenic acid (CGA), a dietary polyphenol, exhibits anti-asthmatic properties, but its precise molecular mechanisms remain poorly understood. This study aimed to elucidate the mechanistic basis of CGA’s anti-asthmatic effects, hypothesizing a central role in regulating polyunsaturated fatty acid metabolism and ferroptosis. An ovalbumin-induced murine asthma model was established in female BALB/c mice to evaluate the therapeutic efficacy of CGA through inflammatory cell counts, cytokine levels (ELISA), and lung histopathology. Integrated lung lipidomics (LC-MS/MS) was employed to profile lipid mediators and phospholipids. The underlying mechanism was investigated in erastin-induced ferroptosis in BEAS-2B cells and validated in mouse lung tissue using qPCR, immunofluorescence, and assays for reactive oxygen species (ROS) and Fe^2+^. CGA treatment significantly attenuated airway inflammation, reduced Th2 cytokine (IL-4, IL-5) and IgE levels, and ameliorated lung pathology in a dose-dependent manner. Lipidomics revealed that asthma was associated with dysregulated docosahexaenoic acid (DHA) metabolism, characterized by elevated pro-inflammatory lipid peroxidation products (e.g., 11-HDoHE, 14-HDoHE), a profile reversed by CGA intervention. Mechanistically, molecular docking and subsequent validation identified CGA as an activator of the Nrf2 antioxidant pathway, leading to upregulation of the key ferroptosis defense genes SLC7A11 and GPX4 both in vitro and in vivo. Consequently, CGA treatment suppressed erastin-induced ROS production and Fe^2+^ accumulation in BEAS-2B cells. In conclusion, this study demonstrates that CGA exerts anti-asthmatic effects by reprogramming DHA metabolism to suppress ferroptosis while enhancing antioxidant pathways. These findings reveal a novel mechanistic axis for CGA and establish that targeting lipid peroxidation-driven ferroptosis represents a promising therapeutic strategy for asthma.

## 1. Introduction

Asthma continues to pose a significant global health burden, characterized by chronic airway inflammation, hyperresponsiveness, and structural remodeling [1,2]. Despite the availability of standard therapies, such as inhaled corticosteroids and β2-agonists, their associated side effects and the emergence of treatment resistance in a substantial proportion of patients highlight the urgent need for novel therapeutic approaches [3,4]. In response, there has been a resurgence of interest in exploring bioactive natural compounds, aiming to leverage their chemical diversity to overcome current therapeutic limitations [5]. Yet, the precise mapping of bioactive constituents and their mechanisms of action remains a major hurdle as it is crucial for both successful clinical translation and rational drug design [6,7].

Chlorogenic acid (CGA), a polyphenolic ester of caffeic and quinic acids, exhibits anti-inflammatory and antioxidant properties [8,9]. Its presence in traditional respiratory medicines supports its potential therapeutic value in asthma [10], and preliminary studies indicate that CGA modulates immune responses and oxidative stress—key processes in asthma pathogenesis [10]. However, current evidence for CGA’s anti-asthmatic effects remains largely descriptive. A lack of mechanistic understanding, including its molecular targets, downstream signaling events, and metabolic consequences, hinders its further therapeutic development [6,11].

CGA was selected for this study for three reasons: (i) it is a representative bioactive component of traditional asthma-related herbs and the metabolite of isochlorogenic acids; (ii) it has been suggested to have anti-asthmatic effects, but a role in ferroptosis has not been tested; and (iii) as a dietary polyphenol, CGA has a well-established safety profile. Natural products are increasingly recognized for restoring disease-related metabolic homeostasis—a concept rooted in negentropy [12], and CGA exemplifies this logic. We therefore propose that CGA’s therapeutic potential in asthma lies in its ability to orchestrate system-level correction of metabolic dysregulation.

The complex interplay between lipid metabolism, oxidative damage, and immune dysregulation in asthma presents a compelling landscape for investigating natural product action [5]. Specifically, asthma is characterized by a profound disturbance in polyunsaturated fatty acid (PUFA) metabolism, particularly of DHA, which fuels a permissive environment for lipid peroxidation [13,14]. This lipotoxic state is a key driver of ferroptosis—an iron-dependent, non-apoptotic cell death pathway increasingly recognized as a central pathological mechanism in chronic inflammatory diseases [15,16]. We therefore hypothesized that CGA exerts its anti-asthmatic effects by reprogramming DHA metabolism to suppress ferroptosis, thereby activating a negentropy loop that quenches airway inflammation and restores tissue integrity [17].

To test this hypothesis, we employed integrated lipidomics and molecular assays using a murine asthma model. The ovalbumin (OVA)-induced mouse model was chosen because it recapitulates key features of human allergic asthma, including Th2-mediated eosinophilic inflammation, elevated IgE levels, and airway hyperresponsiveness. For in vitro mechanistic validation, we used human bronchial epithelial BEAS-2B cells, as airway epithelial cells play a central role in both asthma pathogenesis and ferroptosis. Given the known perturbation of PUFA metabolism in allergic inflammation, integrated lipidomics (LC-MS/MS) was utilized to map asthma-related lipid dysregulation. Our goal was to identify key molecular targets within this pathway, thereby establishing a mechanistic foundation for the development of CGA-based therapeutic interventions.

## 2. Results

### 2.1. Predictive Analysis of Core Targets and Signaling Pathways of CGA Against Asthma

To systematically uncover the potential molecular targets and regulatory networks underlying the anti-asthmatic effects of CGA, this study integrated multiple public databases to retrieve CGA-related targets and asthma-associated disease targets. A multi-dimensional analysis was performed, including protein–protein interaction (PPI) network construction, core target screening, functional enrichment analysis, and molecular docking.

**Figure 1:**
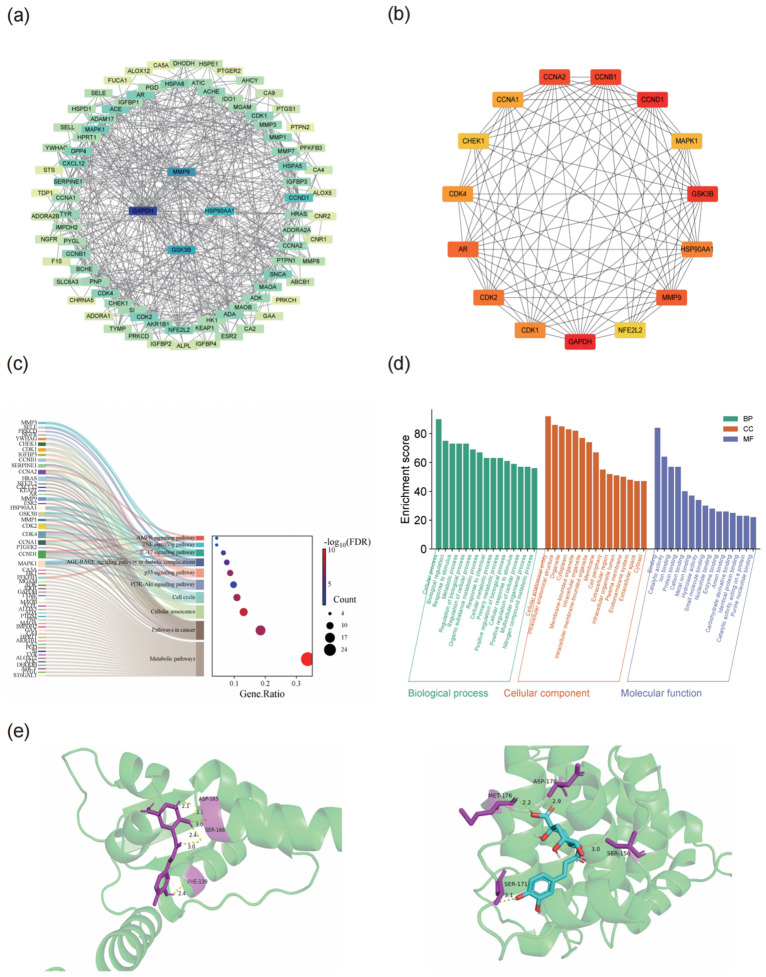
Network pharmacology-based analysis of the anti-asthmatic mechanisms of CGA. (**a**) PPI network of common targets between CGA and asthma. Node color intensity indicates degree centrality. (**b**) Core PPI subnetwork of 15 hub targets (e.g., CCNA2, MAPK1, MMP9, CDK1, NFE2L2). (**c**) KEGG pathway enrichment of core targets. Bubble size represents gene count; color indicates adjusted *p*-value. Key pathways include PI3K-Akt signaling. (**d**) GO functional enrichment (BP, CC, MF) of core targets. (**e**) Molecular docking of CGA with KEAP1 and PI3K. Hydrogen bonds (dashed lines) are formed with residues ASP-165, SER-166, SER-156 (KEAP1) and multiple residues of PI3K.

The overlapping targets between CGA and asthma, identified by Venn diagram analysis, were imported into the STRING database. A PPI network was constructed and visualized using Cytoscape (v3.8.2) (Figure 1a). In this network, nodes represent target proteins and edges denote protein–protein interactions. Topological analysis was conducted to calculate degree, betweenness centrality, and closeness centrality for each node. After removing peripheral proteins, core targets were identified. Nodes with darker colors indicate higher degree values, suggesting more central positions within the network and broader involvement in protein interaction regulation. The network intuitively illustrates the multi-target, multi-pathway regulatory characteristics of CGA against asthma.

Based on topological analysis, peripheral proteins were excluded from the full target network to construct a core PPI subnetwork (Figure 1b). This subnetwork comprised 15 core targets: CCNA2, CCNB1, CCNA1, CCND1, CHEK1, MAPK1, CDK4, GSK3B, AR, HSP90AA1, CDK2, MMP9, CDK1, GAPDH, and NFE2L2. Edges represent protein interactions, and nodes with higher degree values exhibit stronger centrality within the network. Functionally, these core targets are mainly enriched in biological processes critical to asthma pathogenesis, including cell cycle regulation, inflammatory response, matrix remodeling, oxidative stress regulation, and signal transduction, thereby further refining the core regulatory module of CGA against asthma.

To elucidate the signaling pathways involving the core targets, KEGG pathway enrichment analysis was performed using the Metascape platform, and the results were visualized as a bubble plot using the Bioinformatics platform (Figure 1c). The vertical axis represents enriched pathways, while the horizontal axis indicates the gene ratio (i.e., the proportion of target genes enriched in a given pathway). Bubble size reflects the number of enriched targets, and bubble color represents the significance level of enrichment. The results demonstrated that the anti-asthmatic effects of CGA are significantly enriched in the PI3K-Akt signaling pathway, pathways in cancer, and metabolic pathways. Notably, the PI3K-Akt pathway is a central regulator of airway inflammation, airway smooth muscle proliferation and remodeling, and immune responses, suggesting that CGA exerts anti-asthmatic effects through synergistic multi-pathway intervention in various pathological processes, including inflammation, cell proliferation, and immune regulation.

GO functional enrichment analysis of the core targets was conducted using Metascape, covering three categories: biological process (BP), cellular component (CC), and molecular function (MF) (Figure 1d). The BP analysis revealed that the core enriched terms include regulation of apoptosis, inflammatory response, immune response, regulation of cell proliferation, and oxidative stress response, which closely align with the pathological processes of asthma, such as airway inflammation, airway remodeling, and oxidative stress injury. The CC analysis showed that the targets are predominantly localized in the plasma membrane, organelles, and extracellular matrix, suggesting their distribution in key structures for signal transduction and material exchange. The MF analysis identified core enrichments in protein binding, enzyme activity, receptor binding, and catalytic activity. These results systematically delineate the molecular mechanisms of CGA against asthma from a functional perspective.

To validate the direct interaction between CGA and the core anti-asthmatic targets at the molecular level, molecular docking was performed using KEAP1 and PI3K as representative targets, and the results were visualized (Figure 1e). The docking results showed that CGA forms multiple hydrogen bonds with amino acid residues of KEAP1, including ASP-165, SER-166, and SER-156, with bond distances of 2.1 Å, 2.4 Å, and 3.0 Å, respectively. In addition, CGA also formed stable hydrogen bonds with several residues of PI3K. Hydrogen bonds are critical for maintaining stable ligand–receptor binding and enhancing intermolecular affinity. These molecular docking results confirmed the direct binding of CGA to KEAP1 and PI3K. In addition, molecular docking between CGA and ALOX12 was also performed, showing favorable binding affinity. However, molecular docking alone is insufficient to establish ALOX12 as a direct functional target of CGA. Therefore, unlike KEAP1 and PI3K, the involvement of the ALOX12-related pathway in CGA-regulated DHA metabolism remains speculative at this stage, and is based on our lipidomic data, docking analysis (Supplementary Figure S1), and existing literature. Further validation via enzymatic activity assays or loss-of-function experiments is required before concluding a direct interaction.

### 2.2. Effects of CGA on Airway Inflammation and Lung Histopathological Injury in Asthmatic Mice

**Figure 2:**
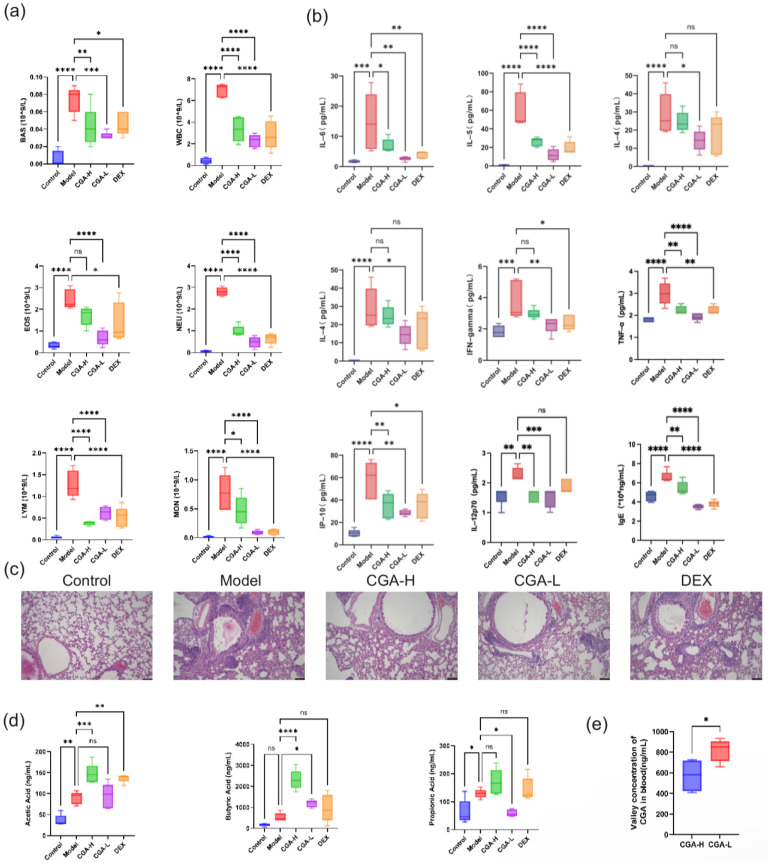
Effects of CGA on airway inflammation, lung histopathology, and plasma concentration in a murine model of asthma. (**a**) Peripheral blood counts of basophils (BASs), eosinophils (EOSs), monocytes (MONs), white blood cells (WBCs), neutrophils (NEUs), and lymphocytes (LYMs) in each group. (**b**) Levels of inflammatory cytokines (IL-5, IL-4, IFN-γ, IL-1β, TNF-α, IL-6, IP-10, IL-12p70) and IgE in bronchoalveolar lavage fluid (BALF). (**c**) Representative hematoxylin and eosin (H&E)-stained lung tissue sections showing histopathological changes (scale bar = 50 μm). (**d**) Fecal levels of short-chain fatty acids (acetate, butyrate, propionate). (**e**) Plasma trough concentrations of CGA in the high-dose (CGA-H) and low-dose (CGA-L) groups. Groups: Control (untreated normal), Model (asthmatic mice), CGA-H (asthmatic mice treated with high-dose CGA), CGA-L (asthmatic mice treated with low-dose CGA), DEX (asthmatic mice treated with dexamethasone, positive control). Data are presented as mean ± SEM. n = 5 per group * *p* < 0.05, ** *p* < 0.01, *** *p* < 0.001, **** *p* < 0.0001; ns, not significant.

To investigate the in vivo anti-inflammatory effects of CGA in the asthma model, inflammatory cell counts in the BALF of mice from each group were first assessed (Figure 2a). The results showed that compared to the normal control group, the numbers of basophils (BASs), eosinophils (EOSs), lymphocytes (LYMs), monocytes (MONs), neutrophils (NEUs), and total white blood cells (WBCs) in the BALF of the asthma model group were significantly increased (*p* < 0.0001). Intervention with both high and low doses of CGA effectively reduced the levels of these inflammatory cells (*p* < 0.01 or *p* < 0.001). Notably, the low-dose CGA group exhibited a more pronounced inhibitory effect on BAS, EOS, LYM, and WBC counts (*p* < 0.01 or *p* < 0.001). The anti-inflammatory effect observed in the dexamethasone (DEX) positive control group was comparable to that of the low-dose CGA group.

Cytokine and immunoglobulin E (IgE) levels in the serum and BALF were subsequently measured (Figure 2b). The levels of pro-inflammatory factors IFN-γ, IL-1β, IL-4, IL-5, IL-6, IP-10, and TNF-α in the BALF were significantly elevated in the asthma model group compared to the normal control group (*p* < 0.01 or *p* < 0.0001). Treatment with both high and low doses of CGA effectively reduced the levels of these pro-inflammatory factors (*p* < 0.05 or *p* < 0.001), with the low-dose group demonstrating a more significant inhibitory effect on IL-1β, IL-5, and IL-6 (*p* < 0.01 or *p* < 0.001). The modulatory effect of the DEX group was similar to that of the CGA-L group. No statistically significant differences were observed among the groups in IL-10, IL-12p70, and IL-13 levels (*p* > 0.05). As a key antibody mediating immediate hypersensitivity reactions, serum IgE levels were significantly higher in the asthma model group compared to the normal control group (*p* < 0.0001), indicating successful model establishment. Following CGA intervention, both high and low doses significantly decreased IgE levels (*p* < 0.01 or *p* < 0.001), with the low dose again showing a more pronounced inhibitory effect (*p* < 0.001). The inhibitory effect on IgE in the DEX group was comparable to that of the low-dose CGA group.

It is noteworthy that OVA-induced allergic asthma is not simply a Th2-dominant inflammatory response. Accumulating evidence has confirmed that Th1 immune disorder is also involved in airway inflammation and airway remodeling in asthma. Chronic allergic asthma frequently presents a bidirectional Th1/Th2 imbalance, rather than absolute activation of a single T helper cell subset [18]. In the present study, the levels of Th1-related IFN-γ and Th2-related IL-4 as well as IL-5 were simultaneously altered after CGA treatment. CGA exerted bidirectional homeostatic regulation on the disrupted Th1/Th2 immune axis, instead of merely inhibiting a single T helper cell subtype. CGA remodeled the overall immune microenvironment, corrected OVA-induced excessive Th2 immune deviation, and moderately strengthened Th1-mediated anti-allergic immune responses, thereby restoring the dynamic balance of Th1/Th2 and alleviating allergic airway inflammation. This mixed cytokine expression pattern is consistent with previous findings of natural polyphenols in the intervention of allergic asthma, and represents a typical immunomodulatory characteristic of plant-derived active ingredients in regulating allergic airway immunity [5].

Finally, the impact of CGA on lung histopathology was evaluated using H&E staining (Figure 2c). Lung tissue from the normal control group displayed intact structure, neatly arranged bronchial mucosal epithelium, and no obvious inflammatory infiltration in the interstitium or alveolar spaces. In contrast, lung tissue from the OVA-induced asthma model group exhibited significant pathological damage, characterized by bronchial and vascular wall injury, narrowed lumens, and extensive peribronchial and perivascular inflammatory cell infiltration. Intervention with both high and low doses of CGA partially reversed this inflammatory damage. Consistent with the other findings, the low-dose CGA group showed a more pronounced reduction in inflammatory cell infiltration and superior restoration of lung tissue architecture. The degree of pathological improvement in the DEX group was comparable to that of the low-dose CGA group. These results further confirm that CGA effectively suppresses asthma-associated airway inflammation and tissue injury, exerting a protective effect on the lung tissue of asthmatic mice.

These pulmonary anti-inflammatory findings suggest a potential involvement of the gut-lung immune axis in CGA-mediated protection against allergic asthma. Combined with our data on fecal acetate, butyrate and propionate profiles (Figure 2d), CGA-induced remodeling of gut microbiota and the resultant changes in SCFA production may contribute to systemic immune modulation, which could further help maintain pulmonary immune homeostasis. Circulating gut-derived SCFAs might improve airway epithelial barrier integrity and modulate the Th1/Th2 immune balance, which may partially account for the bidirectional regulation of pulmonary inflammatory cytokines and pathological injury observed in our study. Notably, high-dose CGA induced greater SCFA elevation, whereas low-dose CGA exhibited superior pulmonary anti-inflammatory efficacy, implying that the protective effect of SCFAs against asthma is not simply concentration-dependent but context-dependent.

### 2.3. Effects of CGA on Short-Chain Fatty Acid and Plasma Drug Concentrations in Asthmatic Mice

The levels of SCFAs in the intestinal contents of the asthmatic mice were measured using GC-MS technology. The results (Figure 2d) showed that, compared to the control group, the levels of acetic acid, propionic acid, and butyric acid in the model group exhibited an increasing trend. Following CGA intervention, the levels of all three SCFAs in the CGA-H group were further elevated compared to the model group. Specifically, the increases in acetic acid and butyric acid were statistically significant (* *p* < 0.001 or ** *p* < 0.0001 vs. the model group), while the change in propionic acid was not (*p* > 0.05). In contrast, SCFA levels in the CGA-L group and the positive control DEX group decreased, falling below or remaining essentially equivalent to those of the model group. The role of SCFAs in asthma is complex and context-dependent. While numerous studies have demonstrated that SCFAs, particularly butyrate and propionate, can enhance airway epithelial barrier function, promote regulatory T cell differentiation, and suppress Th2-driven allergic inflammation [19], the relationship between SCFA levels and asthma severity is not always linear. Our observation that asthmatic mice exhibited elevated SCFA levels compared to controls is consistent with previous studies reporting altered SCFA profiles in the airways of asthmatic patients, where SCFA levels correlated with distinct inflammatory endotypes [20]. Moreover, the further elevation of SCFAs by high-dose CGA may reflect an enhanced compensatory response or a direct modulation of gut microbiota composition by CGA, rather than a direct anti-inflammatory effect. Therefore, the functional significance of SCFA changes in this study warrants further investigation.

Serum drug concentration measurements (Figure 2e) revealed that the trough concentration in the CGA-L group was significantly higher than that in the CGA-H group. This finding aligns with the trend observed in several previous experiments where the low-dose group exhibited stronger modulatory effects, suggesting a non-linear dose-dependency of the in vivo metabolism and pharmacodynamics of CGA. This phenomenon suggests a bifurcated, dose-dependent mechanism: a high oral dose of CGA appears to be preferentially retained and it acts locally within the intestinal lumen, where it significantly promotes gut SCFA (particularly butyrate) production, while a low dose is absorbed more efficiently, yielding higher systemic bioavailability to exert direct anti-inflammatory effects on distal sites like the lung. This complex, non-linear dose–response relationship, potentially governed by the saturation of intestinal efflux transporters or first-pass metabolism at higher doses, provides a compelling mechanistic basis for CGA’s modulation of the “gut-lung axis” in asthma.

### 2.4. CGA Modulates Pulmonary Lipid Mediators in Asthmatic Mice

**Figure 3:**
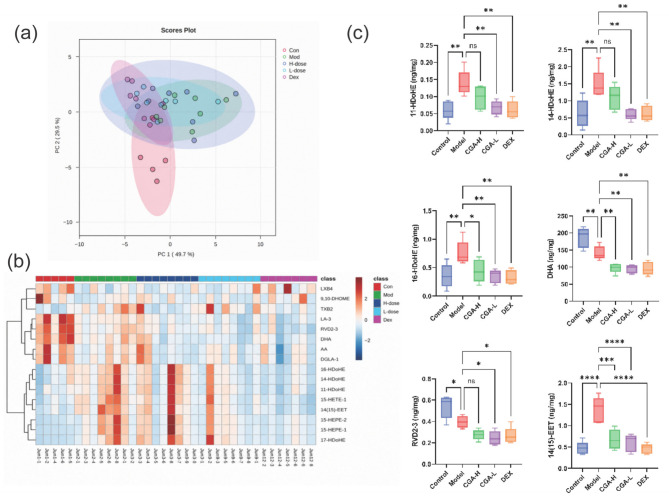
Effects of chlorogenic acid on lipid metabolism (LM) in lung tissues of asthmatic mice. (**a**) Scores plot of lipid metabolites: Metabolic profiles were significantly separated between the control and model groups; the metabolic profile of the low-dose chlorogenic acid group shifted toward the control group. (**b**) Heatmap of lipid metabolites: Lipid peroxidation products were upregulated and DHA was downregulated in the model group; chlorogenic acid reversed the increase in lipid peroxidation products, with the low-dose group showing a more significant effect. (**c**) Targeted quantification of key lipids: Lipid peroxidation products were increased and DHA was decreased in the model group; low-dose chlorogenic acid significantly inhibited lipid peroxidation products, with an effect comparable to that of the DEX group. Data are presented as mean ± SEM. n = 5 per group * *p* < 0.05, ** *p* < 0.01, *** *p* < 0.001, **** *p* < 0.0001; ns, not significant.

To further elucidate the association between the anti-asthmatic effects of CGA and lipid metabolism, this study employed lipidomics analysis (Figure 3a). The results demonstrated a clear separation in the lipid metabolic profiles between the normal control and asthma model groups, indicating that asthma induction led to significant dysregulation of lipid metabolism in mouse lung tissue. Following CGA intervention, the metabolic profile of the low-dose group markedly shifted toward that of the normal control group, whereas the shift in the high-dose group was less pronounced. This suggests that CGA can reverse asthma-associated lipid metabolic abnormalities in a non-monotonic dose-dependent manner.

Heatmap analysis further revealed (Figure 3b) that lipid peroxidation products—including 11-HDoHE, 14(15)-EET, 14-HDoHE, and 16-HDoHE—were significantly upregulated in the asthma model group, while levels of DHA-derived pro-resolving lipid mediators such asDHA and RvD2-3 were markedly downregulated. Intervention with CGA substantially reversed the aberrant accumulation of these pro-inflammatory lipid peroxidation products, with the low-dose group exhibiting a more pronounced regulatory effect. Notably, although CGA exerted obvious anti-inflammatory and anti-asthmatic effects, it further reduced the levels of DHA and RvD2-3. These findings indicate that CGA remodels DHA-related lipid metabolism through a unique regulatory pattern, rather than simply restoring the abundance of pro-resolving lipid mediators. To validate the lipidomics findings, targeted quantification of key lipid metabolites was performed (Figure 3c). The results showed that, compared to the normal control group, the asthma model group exhibited significantly elevated levels of the lipid peroxidation products 11-HDoHE, 14(15)-EET, 14-HDoHE, and 16-HDoHE (*p* < 0.01 or *p* < 0.0001), alongside significantly reduced levels of DHA and RvD2-3 (*p* < 0.05 or *p* < 0.01). Following CGA treatment, the low-dose group significantly reduced the levels of these pro-inflammatory lipid peroxidation products. Although the high-dose group also ameliorated the lipid metabolic disturbances, the effect was weaker than that in the low-dose group. Concurrently, DHA and RvD2-3 levels were further decreased (*p* < 0.05 or *p* < 0.01), an effect comparable to that observed in the DEX group. To interpret this seemingly paradoxical phenomenon, we speculate that CGA may modulate the ALOX12-related pathway, thereby altering the metabolic shunting of DHA toward both pro-inflammatory lipid peroxidation products (e.g., HDoHEs) and pro-resolving mediators (RvD2-3). The beneficial anti-asthmatic effect of CGA may mainly result from the reduction in overall pro-inflammatory lipid peroxidation burden, rather than maintaining the homeostasis of pro-resolving lipid mediators. Additionally, the reduction in inflammatory cell infiltration likely contributed to decreased secretion of lipid mediators such as RvD2-3. Collectively, these observations reflect the precise regulatory effect of CGA on the DHA-centered lipid peroxidation network in asthmatic mice.

### 2.5. Effects of CGA on Pulmonary Phosphatidylcholines in Asthmatic Mice

**Figure 4:**
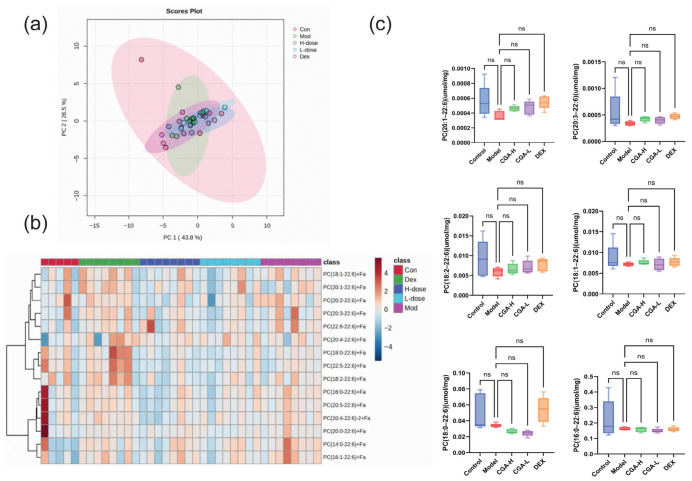
Effects of chlorogenic acid on phosphatidylcholine (PC) metabolism in asthmatic mice. (**a**) Scores plot of PC metabolites in each group. The PC metabolic profiles of the control group and asthma model group showed a separation trend without a statistically significant difference; the metabolic profile of the chlorogenic acid intervention group slightly shifted toward the control group. (**b**) Heatmap of PC metabolites in each group. Polyunsaturated fatty acid-type PCs in the asthma model group showed a downward trend, which was partially reversed after chlorogenic acid intervention with no significant difference. (**c**) Targeted quantitative detection results of PC subtypes in each group. No significant changes were observed in the levels of each PC subtype between the asthma model group and chlorogenic acid intervention group (*p* > 0.05). Data are presented as mean ± SEM. n = 5 per group ns, not significant.

To investigate the regulatory effects of CGA on the metabolic profile of PCs in asthmatic mice, this study employed lipidomics technology combined with targeted quantitative analysis to systematically examine the expression levels of key PC subtypes in lung tissues from different intervention groups (Figure 4).

Lipidomics analysis results (Figure 4a,b) showed that the PC metabolic profiles of the normal control group and the asthma model group exhibited a certain separation trend, although this did not reach statistical significance. Following CGA intervention, the PC metabolic profiles of both the CGA-H and CGA-L groups showed a tendency to shift toward the normal control group; however, their overall clustering characteristics were not markedly different from those of the model group. This suggests that neither asthma induction nor CGA intervention triggered a significant remodeling of the overall PC metabolic landscape. Heatmap analysis further corroborated this trend, revealing a mild downregulation of DHA-containing polyunsaturated PC species in the asthma model group. Although CGA intervention partially reversed the levels of these metabolites to varying degrees, no significant inter-group differences were observed.

Targeted quantitative results further validated the lipidomics findings (Figure 4c). Compared to the normal control group, no significant changes were detected in the levels of core PC subtypes in the lung tissue of the asthma model group (*p* > 0.05). Following intervention with either high or low doses of CGA, the expression levels of each PC subtype remained statistically unchanged compared to the model group (*p* > 0.05). Similarly, the DEX positive control group exhibited no significant alterations in any PC subtype levels. These results demonstrate that, within the asthma model system employed in this study, the basal expression levels of core PC subtypes were not significantly affected by either the pathological progression of asthma or CGA intervention. This suggests that the core anti-asthmatic mechanism of CGA may not depend on the global regulation of PC anabolism and catabolism.

### 2.6. CGA Modulates Ferroptosis-Related Indicators in Human Bronchial Epithelial BEAS-2B Cells

**Figure 5:**
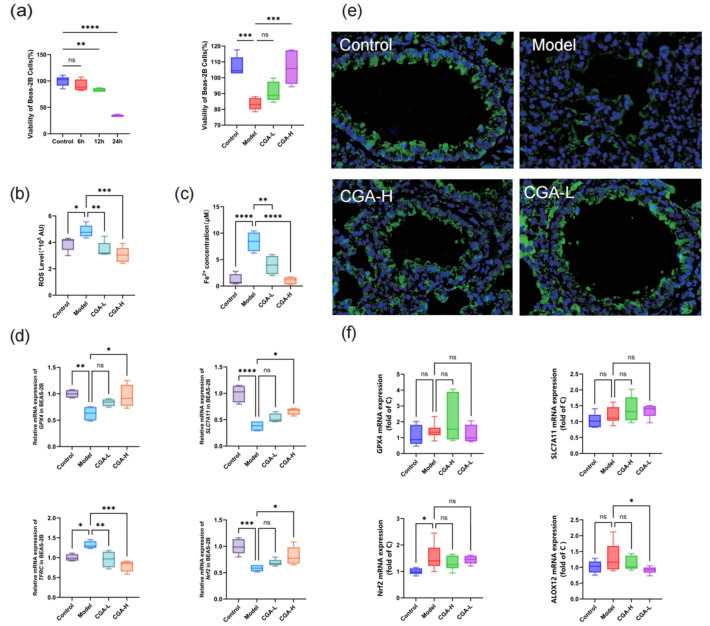
CGA inhibits ferroptosis in BEAS-2B cells and mouse lung tissues. (**a**) CCK-8 assay of BEAS-2B cell viability. (**b**) Detection of lipid ROS levels in BEAS-2B cells. (**c**) Detection of intracellular Fe^2+^ levels in BEAS-2B cells. (**d**) mRNA expression levels of ferroptosis-related genes (GPX4, NRF2, SLC7A11, TFRC) in BEAS-2B cells. (**e**) Immunofluorescence staining of GPX4 protein in mouse lung tissues. Green fluorescence indicates GPX4 protein expression, and blue fluorescence indicates nuclear staining (scale bar = 50 μm). (**f**) mRNA expression levels of ferroptosis-related genes (GPX4, NRF2, SLC7A11, ALOX12) in mouse lung tissues. Data are presented as mean ± SEM. n = 5 per group * *p* < 0.05, ** *p* < 0.01, *** *p* < 0.001, **** *p* < 0.0001; ns, not significant.

To further validate the molecular mechanism underlying the anti-asthmatic effects of CGA, this study established an erastin-induced ferroptosis model in BEAS-2B cells (Supplementary Figure S2) and conducted analyses of cell viability, oxidative stress, iron homeostasis, and expression of genes involved in the key pathways. Cell viability assay results (Figure 5a) demonstrated that, compared to the control group, BEAS-2B cell viability decreased in a time-dependent manner following erastin treatment for 6, 12, and 24 h. Notably, CGA pretreatment effectively reversed the erastin-induced loss of cell viability. Assessment of oxidative stress and iron homeostasis (Figure 5b,c) revealed that, compared to the control group, intracellular ROS levels (*p* < 0.01) and Fe^2+^ accumulation (*p* < 0.0001) were significantly elevated in the erastin-treated group. Following CGA intervention, the high-dose group significantly reduced both ROS levels (*p* < 0.01) and Fe^2+^ accumulation (*p* < 0.001), exhibiting a more pronounced regulatory effect. These findings indicate that CGA effectively alleviates ferroptosis-associated oxidative stress and iron overload.

Subsequent qPCR analysis of ferroptosis-related gene mRNA expression (Figure 5d) showed that, compared to the control group, the model group exhibited significant downregulation of GPX4 and Nrf2 mRNA expression (*p* < 0.01 or *p* < 0.001), alongside significant upregulation of TFRC and SLC7A11 mRNA expression (*p* < 0.0001 or *p* < 0.01). Following CGA intervention, the high-dose group significantly upregulated GPX4 and Nrf2 mRNA expression (*p* < 0.05 or *p* < 0.01) and downregulated TFRC and SLC7A11 mRNA expression (*p* < 0.01 or *p* < 0.0001), with effects superior to those of the low-dose group.

To further validate the regulatory effects of CGA on ferroptosis-related pathways in vivo, animal experiments were conducted (Figure 5e,f). Immunofluorescence staining results (Figure 5e) revealed that, compared to the control group, the fluorescence signal of GPX4 protein in lung tissue was markedly diminished in the asthma model group, indicating significantly reduced GPX4 protein expression. Following CGA intervention, the high-dose group (CGA-H) exhibited substantially enhanced GPX4 fluorescence signals in lung tissue, whereas the recovery in the CGA-L group was relatively limited. These findings demonstrate that CGA effectively upregulates GPX4 protein expression in vivo.

qPCR results (Figure 5f) further validated the regulatory effects of CGA on ferroptosis-related gene mRNA expression. Compared to the control group, the asthma model group showed significantly downregulated GPX4 mRNA expression (*p* < 0.05) and significantly upregulated Nrf2 mRNA expression (*p* < 0.05) in lung tissue. Concurrently, mRNA expression levels of the ferroptosis-related genes SLC7A11 and ALOX12 displayed slight upward trends, although these differences did not reach statistical significance. Following CGA intervention, the high-dose group (CGA-H) significantly upregulated GPX4 mRNA expression (*p* < 0.05). By contrast, SLC7A11 levels only showed mild, non-significant alterations after CGA treatment, with no obvious consistent transcriptional regulation at the mRNA level. These results suggest that CGA exerts anti-ferroptotic and anti-asthmatic effects in vivo mainly by activating the Nrf2-GPX4 antioxidant pathway, while the potential involvement of SLC7A11 and ALOX12 needs further verification at protein and functional levels.

## 3. Discussion

This study elucidates a previously unrecognized mechanism underlying the anti-asthmatic efficacy of chlorogenic acid, centering on the reprogramming of DHA-centered lipid metabolism and the consequent modulation of ferroptosis. We first established that CGA administration significantly alleviated hallmark features of allergic asthma, including airway hyperreactivity, eosinophilic inflammation, and the elevation of Th2 cytokine (IL-4, IL-5, IL-13), and serum IgE levels [21]. This confirmed its potent therapeutic activity, providing the essential phenotypic context for mechanistic investigation. The subsequent lipidomic profiling demonstrated that CGA induces a remodeling of the perturbed DHA-derived lipid mediator network in asthma, an effect that potentially converges on the enzymatic control of lipid peroxidation [22]. Notably, molecular docking analysis predicted a favorable binding affinity between CGA and ALOX12, a key enzyme responsible for oxygenating PUFAs such as DHA to initiate pro-ferroptotic lipid signaling. Combined with the observed reduction in DHA-derived peroxidation products, these computational analyses suggest that CGA may exert its protective effects by modulating the ALOX12-related metabolic axis. Although direct biochemical validation is still required to confirm this inhibitory interaction, our findings indicate that suppression of this lipid-peroxidizing pathway represents a potential upstream regulatory event underlying the anti-asthmatic and anti-ferroptotic actions of CGA [21].

Lipid metabolic reprogramming induced by CGA may be partially mediated by the ALOX12-related cascade, and serves as an important upstream regulatory node underlying its anti-asthmatic and anti-ferroptotic protection. By curbing DHA peroxidation, CGA reduces the cellular burden of lipid hydroperoxides, the primary substrates that drive ferroptosis. This metabolic shift functionally manifested as the marked upregulation of key cellular defense components against ferroptosis. We observed a significant increase in the expression of SLC7A11, a transporter essential for cellular uptake of cystine, the oxidized dimer of cysteine, which is the rate-limiting precursor for glutathione (GSH) biosynthesis [23]. Concurrently, the expression and activity of GPX4, the essential enzyme that utilizes GSH to reduce lipid hydroperoxides to harmless alcohols, were enhanced [24]. This coordinated upregulation of the SLC7A11/GPX4 axis is a canonical adaptive response to counteract ferroptotic stress [25]. The Nrf2 transcription factor, a master regulator of antioxidant responses, is known to transactivate both *SLC7A11* and *GPX4* genes [26]. Our data suggest that CGA may activate the Nrf2 pathway, potentially as a consequence of reduced lipid peroxide load or through direct interaction with KEAP1, leading to this reinforced antioxidant defense system.

**Figure 6:**
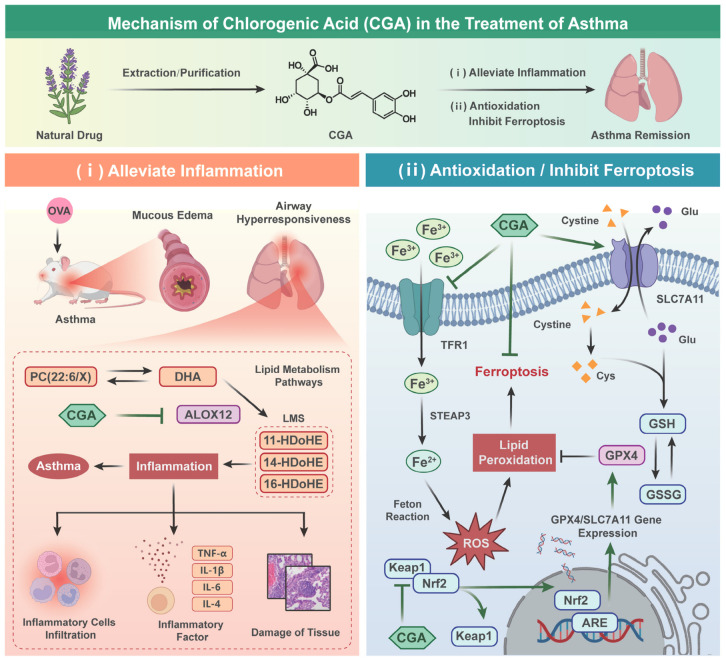
Schematic illustration of the dual mechanisms through which CGA suppresses allergic asthma initiation and recurrence. Pathway 1 (Anti-inflammatory): CGA may regulate ALOX12-related lipid metabolism, remodeling DHA metabolism and reducing DHA-derived lipid mediators (11-HDOHE, 14-HDOHE, 16-HDOHE). This suppresses inflammatory cell infiltration and pro-inflammatory cytokines (TNF-α, IL-1β, IL-6, IL-4), thereby alleviating lung tissue damage. Pathway 2 (Anti-ferroptosis/Antioxidant): CGA directly inhibits Keap1-mediated Nrf2 ubiquitination and promotes Nrf2 stabilization. Nuclear Nrf2 binds ARE to upregulate SLC7A11 (enhancing glutathione synthesis) and GPX4 (inhibiting lipid peroxidation) while concurrently suppressing ROS/iron-mediated ferroptosis.

Based on the integrated findings from this study, a mechanistic model illustrating the anti-asthmatic effects of CGA is presented in Figure 6. In the schematic, CGA exerts its therapeutic activity through a dual, dose-dependent mechanism converging on the regulation of lipid peroxidation and ferroptosis. Molecular docking analysis revealed that CGA directly binds to KEAP1, the principal negative regulator of the Nrf2 pathway [27]. This interaction prevents Nrf2 ubiquitination and subsequent proteasomal degradation, leading to Nrf2 stabilization and nuclear translocation [28]. In the pulmonary compartment, CGA—particularly at a low dose that achieves higher systemic bioavailability—exerts two complementary actions: it may indirectly affect polyunsaturated fatty acid peroxidation through the metabolic network related to ALOX12, a key enzyme initiating the peroxidation of polyunsaturated fatty acids like DHA [29], and it activates the Nrf2 antioxidant pathway via KEAP1 binding [30]. Alteration of the ALOX12-associated metabolic network may mitigate the generation of pro-inflammatory lipid peroxidation products (e.g., 11-HDoHE, 14-HDoHE) [31], while Nrf2 activation subsequently upregulates the expression of the ferroptosis defense genes. Collectively, this study positions CGA as a dual modulator that orchestrates DHA metabolic reprogramming and ferroptosis inhibition through both direct pulmonary KEAP1/Nrf2/ALOX12 signaling and indirect gut-mediated pathways to suppress airway inflammation and remodeling.

Notably, a striking finding of the present study is that the CGA-L group exhibited better therapeutic efficacy than the CGA-H groups, indicating a non-monotonic dose–response relationship. This phenomenon, also referred to as hormesis or the bell-shaped effect, has been documented for other natural antioxidants such as resveratrol and curcumin [32]. Several non-mutually exclusive mechanisms may account for this observation. First, high-dose CGA may cause saturation of intestinal absorption or hepatic metabolism, thereby reducing the bioavailability of the parent compound and its active metabolites and consequently weakening its therapeutic effects. Second, at higher concentrations, CGA may paradoxically induce pro-oxidant effects or activate compensatory stress responses (e.g., upregulation of alternative lipoxygenases or ferroptosis escape pathways), thereby counteracting its intrinsic protective functions [33]. Third, the KEAP1-Nrf2 system, which is modulated by CGA through direct binding, displays concentration-dependent kinetic characteristics; excessive Nrf2 activation may trigger feedback inhibition or target desensitization, impairing its regulatory efficiency [34]. Fourth, the ferroptosis pathway itself involves sophisticated non-linear regulatory nodes [33]. Moderate inhibition of lipid peroxidation exerts protective effects, whereas excessive intervention may disrupt intracellular redox homeostasis, thereby worsening asthma outcomes.

Oxidative stress and lipid peroxidation are well-established contributors to epithelial injury, immune cell activation, and airway remodeling [18]. By targeting ferroptosis, CGA addresses a fundamental cellular damage pathway that fuels inflammation. Although low-dose CGA showed comparable overall efficacy to dexamethasone in reducing airway inflammation and lung pathology, their underlying mechanisms are distinctly different. Our lipidomic data suggest that dexamethasone primarily suppresses phospholipase activity, thereby reducing the release of polyunsaturated fatty acid substrates, rather than directly inhibiting the lipoxygenase-peroxidation-ferroptosis cascade as CGA does. This distinction may underlie differential side-effect profiles and suggests potential for combination therapy or as an alternative to conventional corticosteroids [35].

Although this study delineates a novel mechanism, its translational potential requires further exploration. Pivotal steps include validating the CGA-ALOX12 interaction at the structural level, and bridging our preclinical findings to clinical relevance by assessing efficacy in refractory asthma phenotypes and by addressing pharmacokinetic optimization. Several limitations should be acknowledged: First, the OVA-induced model primarily recapitulates Th2-driven inflammation and may not fully represent human asthma heterogeneity, and the assessment of lung histopathology was not blinded. Second, while our qPCR and immunofluorescence data suggest that CGA activates the Nrf2-GPX4 antioxidant pathway, direct evidence at the protein level remains insufficient, and we will conduct more comprehensive protein analyses in future studies. Third, based on lipidomic data and published evidence, we speculate that CGA modulates DHA metabolism partially through the ALOX12-associated pathway, and molecular docking has been performed to support this hypothesis; however, further validation via enzymatic activity assays or loss-of-function experiments is still needed to confirm the direct interaction and functional relevance. Fourth, as this study was designed primarily for hypothesis generation and mechanistic exploration, the statistical analyses were not adjusted for multiple comparisons. Nevertheless, the consistency of our findings across multiple endpoints and independent experimental approaches supports their robustness. Finally, the present study was conducted in a murine model and in vitro cell lines, and translational studies in humans are needed to assess the clinical relevance of our findings.

In conclusion, our study provides compelling evidence that CGA ameliorates experimental asthma by orchestrating a DHA-centric metabolic reprogramming that quenches ferroptosis [36]. It introduces conceptual innovations on multiple fronts: it redefines the role of CGA as a multi-pathway regulator coordinating DHA metabolic reprogramming and ferroptosis, rather than simply treating it as a direct specific enzyme inhibitor [37]; it integrates the pharmacology of natural compounds with the novel pathophysiology of ferroptosis; and it positions the reprogramming of DHA metabolism as a central therapeutic lever in asthma. This mechanistic elucidation underscores the potential of harnessing specific natural product scaffolds to target novel disease pathways such as lipid peroxidation-driven cell death [38], paving the way for the development of more precise and effective therapeutics for asthma.

## 4. Materials and Methods

### 4.1. Reagents

The following reagents were used in this study. Ultrapure water (mass spectrometry grade, catalog No. W6-4), mass spectrometry-grade methanol (catalog No. A456-4), acetonitrile (catalog No. A955-4), formic acid (catalog Nos. A118P-500, A117-50), isopropanol (catalog No. 67-63-0), and acetic acid (catalog No. A113-50) were obtained from Fisher Scientific (Waltham, MA, USA). Chromatography-grade water (catalog No. 310054GH) was purchased from Wahaha Group (Hangzhou, China). Chromatography-grade n-hexane (catalog No. 8004H-4) was obtained from Concord Technology (Tianjin, China). Ammonium acetate (catalog No. 231401) was purchased from Cleman (Shanghai, China). Anhydrous sodium sulfate (catalog No. S818055) was obtained from Macklin (Shanghai, China). Glycerol (catalog No. SHBM6445) was purchased from Sigma-Aldrich (St. Louis, MO, USA),Butylated hydroxytoluene (BHT, catalog No. 432646) was obtained from Beijing J&K Scientific (Beijing, China). Methyl tert-butyl ether (MTBE, catalog No. 44116-88) was purchased from Honeywell (Morris Plains, NJ, USA). The lipid mediator (LM) internal standard (catalog No. 516671) was purchased from Cayman Chemical (Ann Arbor, MI, USA). The phosphatidylcholine (PC) internal standard (catalog No. 330709W-1EA) was purchased from Avanti Polar Lipids (Alabaster, AL, USA). The ferrous ion assay kit (colorimetric method, catalog No. S1066)was purchased from Beyotime Biotechnology (Shanghai, China). The reactive oxygen species (ROS) assay kit (catalog No. E004) was obtained from Nanjing Jiancheng Bioengineering Institute (Nanjing, China). All other reagents were of analytical or high-performance liquid chromatography grade.

### 4.2. Molecular Docking of Chlorogenic Acid with Core Asthma Targets

To predict the molecular interactions between CGA and key proteins implicated in asthma pathogenesis, a computational molecular docking study was performed. Core target proteins identified as central hubs in asthma-related protein–protein interaction networks were selected as receptors. The three-dimensional (3D) crystal structures of these proteins were retrieved from the RCSB Protein Data Bank (PDB). For targets lacking a resolved crystal structure, homology models were generated using the SWISS-MODEL server. The 3D structure of chlorogenic acid (CID: 1794427) was downloaded in SDF format from the PubChem database and prepared for docking using AutoDock Tools, which included energy minimization and the addition of Gasteiger charges and polar hydrogen atoms.

Molecular docking simulations were conducted using AutoDock Vina software (version 1.1.2). The search space for docking was defined to encompass the entire solvent-accessible surface of each target protein to facilitate blind docking. All other parameters were set to default. For each CGA-target pair, ten independent docking runs were performed. The resulting pose with the most favorable (lowest) predicted binding free energy (ΔG, kcal/mol) was selected for detailed analysis. Specific molecular interactions, such as hydrogen bonds, hydrophobic contacts, and π-π stacking, within the binding complexes were visually inspected and analyzed using the PyMOL Molecular Graphics System (version 2.5.0).

### 4.3. Pharmacodynamic Evaluation of Chlorogenic Acid in an Allergic Asthma Model

#### 4.3.1. Animals and Ethical Statement

Female BALB/c mice (6–8 weeks old, weighing 17–23 g) were housed under controlled conditions (12 h light/dark cycle, 22 ± 2 °C, 55 ± 10% humidity) with ad libitum access to food and water. After purchase, the animals were allowed to acclimatize for 1 week prior to the start of experiments. All experimental protocols were reviewed and approved by the Institutional Animal Care and Use Committee (IACUC) of Xinjiang Medical University (Protocol No.: XJIMM-20230704; date of approval: 4 July 2023) and were conducted in strict accordance with the National Institutes of Health Guide for the Care and Use of Laboratory Animals.

#### 4.3.2. Asthma Model Induction and Experimental Design

A total of 38 female BALB/c mice were used in this study. Mice were randomly assigned to five groups (n = 7–8 per group): (1) Normal control (NC), (2) Asthma model (OVA), (3) CGA high-dose (CGA-H, 20 mg/kg), (4) CGA low-dose (CGA-L, 10 mg/kg), and (5) Dexamethasone (DEX). After data collection, the highest and lowest values in each group were excluded as outliers based on a predefined criterion to minimize the influence of extreme values. Thus, 5 mice per group were included in the final analysis (n = 5 for all groups). No other animals or data points were excluded.

Allergic asthma was induced via ovalbumin (OVA, Grade V, Sigma-Aldrich) sensitization and challenge. On day 0 and day 8, mice (except the NC group) were intraperitoneally injected with 0.2 mL of a suspension containing 25 µg of OVA adsorbed onto 2 mg of aluminum hydroxide (Al(OH)_3_) gel in sterile saline. The NC group received saline alone. From day 15 to day 21, mice were subjected to daily 30-min aerosol challenges with 1% (*w*/*v*) OVA in saline using an ultrasonic nebulizer. One hour prior to each challenge (days 15–21), the CGA-H and CGA-L groups received their respective treatments via oral gavage. The NC and OVA model groups received an equivalent volume of vehicle (saline).

#### 4.3.3. Sample Collection and Processing

Twenty-four hours after the final challenge, mice were anesthetized. Blood was collected via the retro-orbital plexus, and serum was isolated by centrifugation (3000× *g*, 15 min, 4 °C) and stored at −80 °C. Bronchoalveolar lavage fluid (BALF) was obtained by cannulating the trachea and lavaging the lungs three times with 0.5 mL of ice-cold PBS. The BALF was centrifuged (2000× *g*, 10 min, 4 °C); the cell pellet was resuspended for differential cell counting using a hemocytometer after Wright-Giemsa staining, and the supernatant was stored at −80 °C for cytokine analysis. Lung tissues were harvested: the left lobe was fixed in 4% paraformaldehyde for histology, and the right lobe was snap-frozen in liquid nitrogen for subsequent molecular analyses.

#### 4.3.4. Histopathology and Cytokine Measurement

Fixed lung tissues were paraffin-embedded, sectioned (5 µm), and stained with hematoxylin and eosin (H&E). Pathological changes, including inflammatory cell infiltration and airway remodeling, were evaluated under a light microscope. The concentrations of interleukin (IL)-4, IL-5, IL-6, tumor necrosis factor-alpha (TNF-α), and interferon-gamma (IFN-γ) in BALF supernatant, and of immunoglobulin E (IgE) in serum were quantified using ELISA kits purchased from Cusabio Biotech Co., Ltd. (Wuhan, China).

### 4.4. Mechanistic Investigation via Targeted and Untargeted Lipidomics

#### 4.4.1. Targeted Analysis of Lipid Mediators (LMs) in Lung Tissue

Approximately 5 mg of frozen lung tissue was homogenized in 175 µL of ice-cold acetonitrile containing 0.1% butylated hydroxytoluene (BHT). After centrifugation (13,300× *g*, 10 min, 4 °C), 100 µL of supernatant was processed with deuterated internal standards (e.g., d8-5-HETE, d4-LTB4) and subjected to solid-phase extraction (SPE) using a Waters Oasis MAX 96-well plate purchased from Waters Corporation (Milford, MA, USA). LMs were eluted, dried under nitrogen, and reconstituted in 50 µL acetonitrile/methanol (50:50, *v*/*v*) for LC-MS/MS analysis.

Quantification was performed using an Agilent 1290 UHPLC system coupled to an Agilent 6495C triple quadrupole mass spectrometer (Agilent Technologies, Santa Clara, CA, USA) operating in negative electrospray ionization (ESI-) mode with multiple reaction monitoring (MRM). Separation was achieved on an Acquity UPLC BEH C18 column (Waters Corporation, Milford, MA, USA). Data were processed using Agilent MassHunter software, version 13.0 (Agilent Technologies, Santa Clara, CA, USA).

#### 4.4.2. Untargeted Analysis of Phospholipids (PCs)

The residual pellet from LM extraction was further processed to obtain a comprehensive lipidomic profile. Lipids were extracted with methanol and methyl tert-butyl ether (MTBE). The organic phase was dried and reconstituted in 50 µL of acetonitrile/isopropanol (50:50, *v*/*v*).

Analysis was conducted using a Thermo Scientific Vanquish UHPLC system coupled to a Q Exactive HF Orbitrap mass spectrometer (Thermo Fisher Scientific, Waltham, MA, USA). Data were acquired in both positive and negative ionization modes with data-dependent MS/MS. Lipid identification and semi-quantification were performed using LipidSearch software, version 5.2 (Thermo Fisher Scientific, Waltham, MA, USA).

#### 4.4.3. Analysis of Short-Chain Fatty Acids (SCFAs) in Intestinal Content

Approximately 10 mg of intestinal content was homogenized in 0.5% sulfuric acid solution. After extraction with MTBE and dehydration with anhydrous sodium sulfate, the organic phase was analyzed by gas chromatography-mass spectrometry (GC-MS) using an Agilent 7890B/5977B system (Agilent Technologies, Santa Clara, CA, USA) equipped with a DB-FFAP column (Agilent Technologies, Santa Clara, CA, USA). Quantification was performed using selective ion monitoring (SIM) mode with external calibration standards.

### 4.5. Molecular Mechanism Exploration: Focus on Ferroptosis Inhibition

#### 4.5.1. Cell Culture and Treatment

Human bronchial epithelial cells (BEAS-2B, ATCC^®^ CRL-9609™) were maintained in DMEM supplemented with 10% fetal bovine serum and 1% penicillin-streptomycin at 37 °C with 5% CO_2_. To induce ferroptosis, cells were treated with 1 µM Erastin (MedChemExpress (MCE), Monmouth Junction, NJ, USA) for 12 h (Supplementary Figure S2). For intervention studies, cells were pre-treated with Erastin for 12 h, followed by co-treatment with chlorogenic acid (20 µM or 40 µM) for an additional 12 h. Control groups included untreated cells and Erastin-only treated cells.

#### 4.5.2. Cell Viability Assay

Cell viability was assessed using the Cell Counting Kit-8 (CCK-8, Beyotime Biotechnology, Shanghai, China). Cells were seeded in 96-well plates, treated as indicated, and then incubated with CCK-8 reagent for 2 h. Absorbance was measured at 450 nm using a microplate reader (BioTek Instruments, Inc., Winooski, VT, USA).

#### 4.5.3. Measurement of Intracellular Reactive Oxygen Species (ROS)

Intracellular ROS levels were detected using the fluorescent probe DCFH-DA (Beyotime Biotechnology, Shanghai, China). After treatment, cells were incubated with 10 µM DCFH-DA for 30 min at 37 °C in the dark. Fluorescence intensity was measured by flow cytometry (excitation/emission: 488/525 nm) and normalized to the total protein content determined by a BCA assay.

#### 4.5.4. Quantification of Intracellular Fe^2+^

The level of ferrous iron (Fe^2+^) was measured using a colorimetric Iron Assay Kit (Beyotime Biotechnology, Shanghai, China) following the manufacturer’s protocol. Absorbance was read at 593 nm, and Fe^2+^ concentration was calculated based on a standard curve and normalized to protein concentration.

#### 4.5.5. Quantitative Real-Time PCR (qPCR)

Total RNA was extracted from BEAS-2B cells using TRIzol reagent. RNA quality and concentration were verified spectrophotometrically. First-strand cDNA was synthesized using a HiFiScript cDNA Synthesis Kit (CWBIO, Taizhou, China). Quantitative PCR was performed on a QuantStudio 6 Flex system using SYBR Green chemistry (CWBIO, Taizhou, China). The relative mRNA expression levels of target genes related to ferroptosis (*GPX4*, *NRF2*, *SLC7A11*, *TFRC*) were calculated using the 2^–ΔΔCt^ method, with *GAPDH* serving as the endogenous control. Primer sequences are shown in Table 1.

### 4.6. Data Processing and Statistical Analysis

Lipidomics data were analyzed using MetaboAnalyst 6.0 based on VIP scores (>1) and independent-sample *t*-test. Other data were analyzed using GraphPad Prism 9.0 and are presented as mean ± standard error of the mean (SEM). Normality was assessed using the Shapiro–Wilk test, and homogeneity of variances was assessed using Levene’s test. Two-group comparisons were performed using unpaired two-tailed Student’s *t*-test, and multi-group comparisons were performed using one-way ANOVA with Tukey’s post hoc test. When parametric assumptions were violated, the Kruskal–Wallis test with Dunn’s post hoc test was used. All experiments were performed with at least three biological replicates per group; for the main experiments, n = 5 per group. Statistical significance was defined as *p* < 0.05. Significance levels are indicated as * *p* < 0.05, ** *p* < 0.01, *** *p* < 0.001, **** *p* < 0.0001; ns indicates not significant (*p* ≥ 0.05).

## Figures and Tables

**Table 1:** PCR primer sequences.

Gene	Primer Sequence
*GAPDH*	Forward: GGAGTCCACTGGCGTCTTCA Reverse: GTCATGAGTCCTTCCACGATACC
*NRF2*	Forward: TCCAGTCAGAAACCAGTGGAT Reverse: GAATGTCTGCGCCAAAAGCTG
*GPX4*	Forward: CCCGATACGCTGAGTGTGGTTTG Reverse: TCTTCGTTACTCCCTGGCTCCTG
*SLC7A11*	Forward: TGCCCTTTCCCTCTATTCGG Reverse: TAATGTTCTGGTTATTTTCTCCGAC
*TFRC*	Forward: GGACGCGCTAGTGTTCTTCT Reverse: CATCTACTTGCCGAGCCAGG

## Data Availability

Data are unavailable due to privacy or ethical restrictions.

## Supplementary Materials

The following supporting information can be downloaded at: https://www.mdpi.com/article/10.3390/ijms27114747/s1.

## Abbreviations

The following abbreviations are used in this manuscript:

CGA	Chlorogenic acid
CGA-L	Chlorogenic acid low-dose (10 mg/kg)
CGA-H	Chlorogenic acid high-dose (20 mg/kg)
DHA	Docosahexaenoic acid
PUFA	Polyunsaturated fatty acid
OVA	Ovalbumin
ELISA	Enzyme-linked immunosorbent assay
LC-MS/MS	Liquid chromatography-tandem mass spectrometry
ROS	Reactive oxygen species
Nrf2/NFE2L2	Nuclear factor erythroid 2-related factor 2
GPX4	Glutathione peroxidase 4
SLC7A11	Solute carrier family 7 member 11
ALOX12	Arachidonate 12-lipoxygenase
KEAP1	Kelch-like ECH-associated protein 1
PI3K	Phosphoinositide 3-kinase
BALF	Bronchoalveolar lavage fluid
H&E	Hematoxylin and eosin
SCFA	Short-chain fatty acid
PC	Phosphatidylcholine
DEX	Dexamethasone
Th2	T helper type 2
IgE	Immunoglobulin E
IL	Interleukin
TNF-α	Tumor necrosis factor alpha
IFN-γ	Interferon-gamma
IP-10	Interferon gamma-induced protein 10
CCK-8	Cell Counting Kit-8
qPCR	Quantitative real-time polymerase chain reaction
GAPDH	Glyceraldehyde-3-phosphate dehydrogenase
TFRC	Transferrin receptor
BHT	Butylated hydroxytoluene
MTBE	Methyl tert-butyl ether
GC-MS	Gas chromatography-mass spectrometry
SPE	Solid-phase extraction
MRM	Multiple reaction monitoring
PPI	Protein–protein interaction
KEGG	Kyoto Encyclopedia of Genes and Genomes
GO	Gene Ontology
BP	Biological process
CC	Cellular component
MF	Molecular function
BASs	Basophils
EOSs	Eosinophils
LYMs	Lymphocytes
MONs	Monocytes
NEUs	Neutrophils
WBCs	White blood cells
HDoHE	Hydroxydocosahexaenoic acid
EET	Epoxyeicosatrienoic acid
RvD	Resolvin D
CCNA2	Cyclin A2
CCNB1	Cyclin B1
CCND1	Cyclin D1
CDK	Cyclin-dependent kinase
MAPK1	Mitogen-activated protein kinase 1
MMP9	Matrix metallopeptidase 9
GSK3B	Glycogen synthase kinase 3 beta
HSP90AA1	Heat shock protein 90 alpha family class A member 1
AR	Androgen receptor
