# Supplementary material for: Chlorogenic Acid Alleviates Experimental Asthma by Reprogramming DHA Metabolism to Inhibit Ferroptosis
Source: Int J Mol Sci. 2026 May 25;27(11):4747. doi: 10.3390/ijms27114747 (PMC13256434; doi:10.3390/ijms27114747)
Supplement: Supplementary file 1 [file ijms-27-04747-s001.zip › ijms-4304275-supplementary.pdf]

Supplementary Information 1:

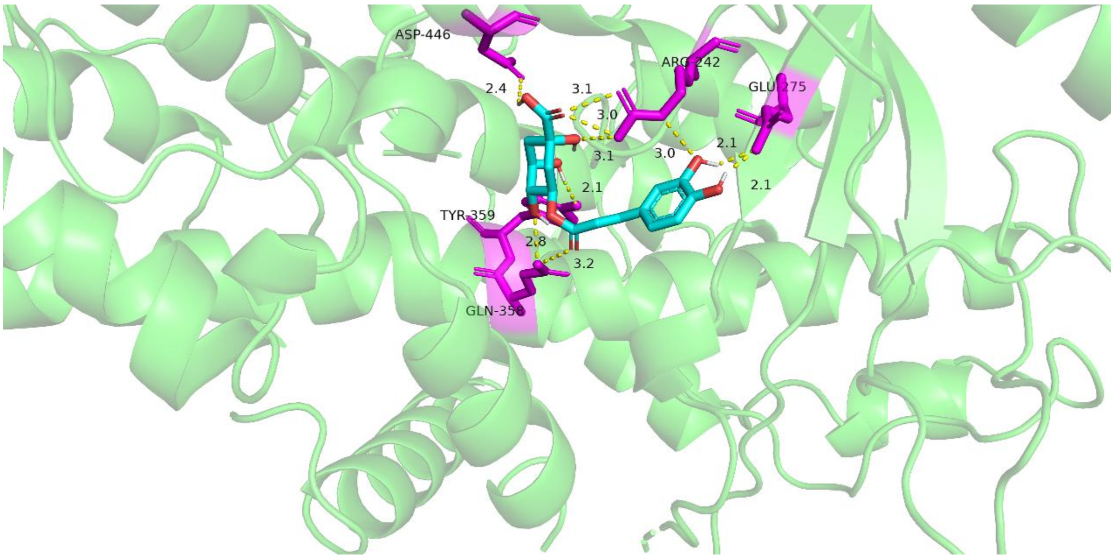

Supplementary figure S1 Molecular docking analysis of chlorogenic acid (CGA) with ALOX12 Molecular docking predicts that CGA binds within the putative active site of ALOX12, forming hydrogen bonds or hydrophobic interactions with several key amino acid residues, including ASP-446, TYR-359, GLN-350, ARG-42, and GLU-275, with calculated interaction distances ranging from 2.1 to 3.2 Å. These computational predictions provide a structural basis for the hypothesis that CGA may directly modulate ALOX12 activity, thereby influencing DHA peroxidation and the downstream ferroptosis signaling pathway (see main text, Figure 6)

Supplementary Information 2:

Regarding the working concentration and treatment duration of erastin used to establish the in vitro ferroptosis model, we primarily determined the concentration range based on previously published literature and further established the optimal modeling conditions in combination with our preliminary experimental results. Due to space and formatting limitations of the main manuscript, the relevant pre-screening process and literature basis are not presented in the main text. We will provide two key screening results in the supplementary materials (see accompanying figures) to make the modeling conditions more complete, transparent, and reproducible.

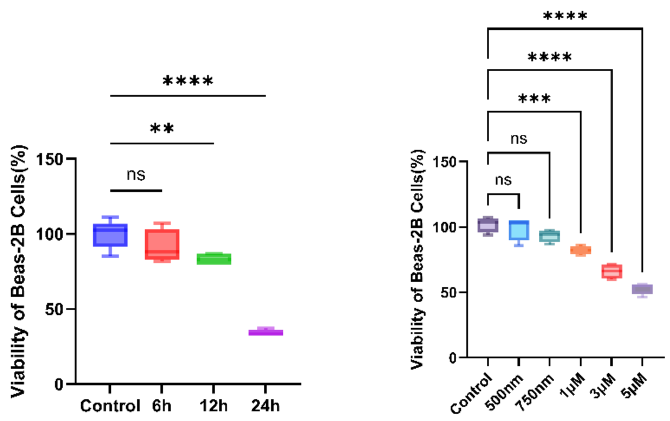

Supplementary figure S2 Optimization of erastin treatment conditions for establishing the ferroptosis model in BEAS-2B cells. Left: Time-course analysis of BEAS-2B cells treated with a fixed concentration of erastin. Right: Dose-response analysis of BEAS-2B cells treated with indicated concentrations of erastin for 12 hours. Cell viability was measured for both panels. Data are shown as mean ± SD (n=5). $^{*}p < 0.05$, $^{**}p < 0.01$ vs. control.
